# Defining the molecular role of gp91phox in the immune manifestation of acute allergic asthma using a preclinical murine model

**DOI:** 10.1186/1476-7961-10-2

**Published:** 2012-01-04

**Authors:** Ena Ray Banerjee, William R Henderson

**Affiliations:** 1 Department of Medicine, Division of Allergy and Infectious Diseases, Center for Allergy and Inflammation, University of Washington, Room 254, 815 Mercer Street, Seattle, WA 98195, USA; 2 Associate Professor, Dept. of Zoology, University of Calcutta, 35, Ballygunge Circular Road, Kolkata- 700019, West Bengal, India

## Abstract

**Objective:**

The phenomena manifested during inflammation require interplay between circulating effector cells, local resident cells, soluble mediators and genetic host factors to establish, develop and maintain itself. Of the molecues involed in the initiation and perpetuation of acute allergic inflammation in asthma, the involvement of effector cells in redox reactions for producing O_2_^- ^(superoxide anion) through the mediation of NADPH oxidase is a critical step. Prior data suggest that reactive oxygen species (ROS) produced by NADPH oxidase homologues in non-phagocytic cells play an important role in the regulation of signal transduction, while macrophages use a membrane-associated NADPH oxidase to generate an array of oxidizing intermediates which inactivate MMPs on or near them.

**Materials and Methods and Treatment:**

To clarify the role of gp91phox subunit of NADPH oxidase in the development and progression of an acute allergic asthma phenotype, we induced allergen dependent inflammation in a gp91*^phox^*-/- single knockout and a gp91phox-/-MMP-12-/- double knockout mouse models.

**Results:**

In the knockout mice, both inflammation and airway hyperreactivity were more extensive than in wildtype mice post-OVA. Although OVA-specific IgE in plasma were comparable in wildtype and knockout mice, enhanced inflammatory cell recruitment from circulation and cytokine release in lung and BALf, accompanied by higher airway resistance as well as Penh in response to methacholine, indicate a regulatory role for NADPH oxidase in development of allergic asthma. While T cell mediated functions like Th2 cytokine secretion, and proliferation to OVA were upregulated synchronous with the overall robustness of the asthma phenotype, macrophage upregulation in functions such as proliferation, and mixed lymphocyte reaction indicate a regulatory role for gp91phox and an overall non-involvement or synergistic involvement of MMP12 in the response pathway (comparing data from gp91phox-/- and gp91phox-/-MMP-12-/- mice).

## Introduction

Asthma is a complex syndrome with well described pathology. However, animal and clinical studies in humans continue to provide conflicting data on contribution of local cells viz. airway epithelial, endothelial and smooth muscle cells, fibroblasts etc vs. cells recruited from circulation. Asthma is characterized by accumulation of inflammatory cells in the lung and airways, secretion of predominantly Th2 cytokines in the lung and airways, epithelial desquamation, goblet cell hyperplasia, mucus hypersecretion and thickening of submucosa resulting in bronchoconstriction and airway hyperresponsiveness. Dysregulated immunity seems to suppress Th1 response and triggers Th2 response whose development is promoted by antigen presenting cells. Th2 cytokines (IL-4, 5, 9, 13) from these cells of which IL-4 and 13 promote B cell differentiation into plasma cells that secrete IgE. Cross-linking of IgE receptors on mast cell sreleases histamines, prostaglandins, thromboxane and leukotrienes leading to bronchoconstriction, vasodilation and mucus secretion. A cascade of interactions between cells and soluble molecules result in bronchial mucosal inflammation and lead to airway hyperresponsiveness.

The production of superoxide anions (O_2_^-^) by neutrophils and other phagocytes is an important step in our body's innate immune response. O_2_^- ^is the precursor of a range of chemicals generally referred to as ROS (reactive oxygen species) [1]. These act as microbicidal agents and kill invading micro-organisms either directly or through the activation of proteases [2,3]. O_2_^- ^is produced by the NADPH oxidase, a multi-protein enzyme complex, which is inactive in resting phagocytes, but becomes activated after interaction of the phagocyte with pathogens and their subsequent engulfment in the phagosome [4]. Defects in the function of the NADPH oxidase result in a severe immunodeficiency, and individuals suffering from CGD (chronic granulomatous disease), a rare genetic disorder that is caused by mutations in NADPH oxidase genes, are highly susceptible to frequent and often life-threatening infections by bacteria and fungi [5]. The microbicidal activity of ROS has generally been seen as the only beneficial function of these chemicals, and uncontrolled production of ROS has been implicated in tissue destruction and a number of disease states. However, over the last couple of years, it has become apparent that ROS produced by NADPH oxidase homologues in non-phagocytic cells also play an important role in the regulation of signal transduction, often via modulation of kinase and phosphatase activities or through gene transcription [6]. These NADPH oxidase homologues are referred to as Nox enzymes (gp91^phox ^is specified as Nox2; where phox is phagocytic oxidase), and several members of this novel protein family have been identified so far.

There is increasing evidence that redox regulation of transcription, particularly activator protein-1 (AP-1) and nuclear factor kappa B (NF-κB), is important in inflammatory diseases. NADPH oxidase, the primary source of reactive oxygen species is a strong candidate for the development of therapeutic agents to ameliorate inflammation and end-organ damage. The possibility of gene therapy for inherited diseases with a single gene mutation had been verified by the successful treatment with bone marrow transplantation. As the gene therapy method and theory has been progressing rapidly, it is expected that gene therapy will overcome the complications of bone marrow transplantation. Of these inherited diseases, chronic granulomatous disease (CGD) is one of the most expected disease for gene therapy. CGD is an inherited immune deficiency caused by mutations in any of the following four phox genes encoding subunits of the superoxide generating phagocyte NADPH oxidase. It consists of membranous cytochromeb558 composed of gp91 phox and p22 phox, and four cytosolic components, p47 phox, p67 phox, rac p21 and p40 phox, which translocate to the membrane upon activation. The gp91^phox ^subunit (also called the β-subunit of the cytochrome) consists of 570 amino acids and has a molecular mass of 65.3 kDa, but runs as a broad smear of approx. 91 kDa on SDS/polyacrylamide gels due to a heterogeneous glycosylation pattern of three asparagine residues (Asn^132^, Asn^149 ^and Asn^240^) [7]

The N-terminal 300 amino acids are predicted to form six transmembrane α-helices, while the C-terminal cytoplasmic domain contains the binding sites for FAD and NADPH, shown experimentally through cross-linking studies and the observation that relipidated flavocytochrome alone can generate O_2_^- ^. In addition, gp91^phox ^is responsible for complexing the two non-identical haem groups of the NADPH oxidase via two histidine pairs. Hence gp91^phox ^contains all co-factors required for the electron transfer reaction which occurs in two steps. First, electrons are transferred from NADPH on to FAD and then to the haem group in the second step to reduce O_2 _to O_2_^- ^in a one-electron-transfer reaction. At present, no information is available on the three-dimensional structure of gp91^phox ^or fragments thereof, although a model for the structure of the cytoplasmic domain of gp91^phox ^has been suggested based on sequence homology with the FNR (ferredoxin-NADP reductase) family [8]. Significant insight into the topology of the cytochrome and the sites of interaction with other oxidase components has been gained through the use of a number of techniques, including epitope mapping or random sequence peptide phage analysis. Additionally, the study of cytochrome isolated from patients with X-linked CGD has contributed to our current understanding of its function [9].

Involvement of the gp91phox subunit in oxidative burst response by PMNs as well as Mϕs is not clear. Macrophages use a membrane-associated NADPH oxidase to generate an array of oxidizing intermediates. In some studies, it has been demonstrated that oxidants potently and efficiently inactivate matrilysin (MMP-7) by cross-linking adjacent tryptophan-glycine residues within the catalytic domain of the enzyme. These *in vitro *observations suggest that MMP inactivation can occur on or near phagocytes that produce both MMPs and reactive intermediates. In the absence of reactive intermediates, unrestrained proteolytic activity might lead to detrimental tissue damage. Indeed, inherited deficiency of gp91*^phox^*, a phagocyte-specific component of the NADPH oxidase required for oxidant production, and targeted deletion of its mouse homologue result in granuloma formation and excessive tissue destruction [10]. Aberrant regulation of MMP activity may contribute to the damage that occurs when phagocytes are unable to generate oxidants. *gp91^phox ^*mutant mice were found to develop spontaneous, progressive emphysema, equal to that seen in smoke-exposed wild-type animals. Macrophages and neutrophils use membrane-associated NADPH oxidase to generate reactive oxygen intermediates. The initial product of the NADPH oxidase is superoxide, which is converted into oxidizing oxygen and nitrogen species. Because their characteristic end products have been detected in diseases ranging from atherosclerosis to neurodegenerative disorders, reactive oxygen and nitrogen intermediates are thought to contribute to inflammatory tissue injury. However, humans and animals deficient in phagocyte NADPH oxidase tend to form granulomas and to have excessive tissue destruction and an exuberant inflammatory response, raising the possibility that oxidants derived from white cells actually govern or suppress inflammation.

One potential mechanism whereby reactive oxygen species can influence inflammation and the associated tissue damage is by regulating the activity of MMPs. In addition to their ability to act on extracellular matrix, MMPs can affect inflammation by directly or indirectly regulating the activity of inflammatory mediators such as chemokines. Because reactive intermediates effectively inactivate MMPs *in vitro*, they provide an efficient mechanism for inhibiting unregulated catalysis by these extracellular proteinases, thereby preventing pathological destruction of tissue proteins and exuberant inflammation. Production of reactive intermediates by the phagocyte NADPH oxidase could confine MMP activity in space and time, permitting only bursts of pericellular proteolysis.

This study endeavours to address the relationship between gp91phox and MMP in the onset and establishment of acute allergic asthma in a mouse model using genetic knockout mice, gp91phox-/- which will be referred to as NOX-/- and MMP12NOX double knockout.

## Materials and methods

### Mice

Both gp91^phox-/- ^mice [11] (Jackson Laboratories, Bar Harbor, ME) and mmp12^-/- ^mice [12] were on a C57Bl/6J background and had been outcrossed and then intercrossed for three generations to generate animals deficient in both genes. C57BL6 mice (Taconic) were used as the control group and are called wildtype. In total the following number of animals were used in each group: n for Control groups were WT (wildtype) 14, NOX-/- (gp91phox -/-) 14, DKO (double knockout MMP12NOX-/-) 16 and n for OVA treated groups were respectively 16, 15, and 14. Mortality was less than 1%. Animals were maintained under strict SPF conditions following guidelines set down by the University of Washington IUCAC under protocol numbers 21-6404 and 2437-05.

### Allergen sensitization and challenge

Mice were sensitized and later challenged with OVA (Pierce, Rockford, IL) as described previously [13,14]. Mice were immunized with OVA (100 μg) complexed with aluminium sulfate in a 0.2-ml volume, administered by i.p. injection on day 0. On days 8 (250 μg of OVA) and on days 15, 18, and 21 (125 μg of OVA), mice were anesthetized briefly with inhalation of isoflurane in a standard anesthesia chamber and given OVA by intratracheal (i.t.) administration. Intratracheal challenges were done as described previously (Iwata A, JI, 2001). Mice were anesthetised and placed in a supine position on the board. The animal's tongue was extended with lined forceps and 50 μl of OVA(in the required concentration) was placed at the back of its tongue. The control goup received normal saline with aluminium sulfate by i.p. route on day 0 and 0.05 ml of 0.9% saline by i.t. route on days 8, 15, 18, and 21.

### Pulmonary function test

*In vivo *airway hyperresponsiveness to methacholine was measured 24 hours after the last OVA challenge by both invasive and non-invasive plethysmography.

### Invasive plethysmography

On d 22, 24 h after the last intra-tracheal allergen (OVA) challenge invasive pulmonary mechanics were measured in mice in response to methacholine in the same manner as previously described [13]with the following modifications: a) the thorax was not opened, b) mice were ventilated with a tidal volume of 200 μl and respiratory rate of 120 breaths/min using a MiniVent Ventilator for Mice (Harvard Apparatus, Holliston, MA), c) mice received aerosolized solutions of methacholine (0, 3.125, 6.25, 12.5, 25, 50, and 100 mg/ml in normal saline) via an AER 1021 nebulizer aerosol system (Buxco Electronics, Inc., Wilmington, NC) with 2.5-4 micron aerosol particle size generated by NEB0126 nebulizer head (Nektar Therapeutics, San Carlos, CA), and d) a commercial plethysmography system (Model PLY4111 plethysmograph, MAX II amplifier and pressure transducer system, and Biosystem XA software, Buxco Electronics, Inc.) was used to determine R_L _as calculated from measures of pressure and flow and expressed as cmH_2_O/ml/s). Non-invasive plethysmography (expressed as Penh) was also assessed on d 22 in independent experiments.

***Non-invasive whole body plethysmography ***in conscious, free moving, spontaneously breathing mice using whole-body plethysmography (model PLY 3211; Buxco Electronics, Sharon, CT) as previously described [14]Mice were challenged with aerosolized saline or increasing doses of methacholine (5, 20, and 40 mg/ml) generated by an ultrasonic nebulizer (DeVilbiss Health Care, Somerset, PA) for 2 min. The degree of bronchoconstriction was expressed as enhanced pause(P_enh_), a calculated dimensionless value, which correlates with the measurement of airway resistance, impedence, and intrapleural pressure in the same mouse. P_enh _readings were taken and avergared for 4 min after each nebulization challenge. Penh was calculated as follows: P_enh _= [(Te/Tr-1)X (PEF/PIF), where T_e _is expiration time, T_r _is relaxation time, PEF is peak expiratory flow, and PIF is peak inspiratoy flow × 0.67 co-efficient. The time for the box pressure to change from a maximum to a user-defined percentage of the maximum represents the relaxation time. The T_r _measurement begins at the maximum box pressure and ends at 40%.

### BAL

After pulmonary function testing, the mouse underwent exsanguination by intra-orbital arterial bleeding and then BAL (0.4 ml three times) of both lungs. Total BAL fluid cells were counted from a 50 μl aliquot and the remaining fluid was centifuged at 200 *g *for 10 min at 4°C and the supernatants stored at -70°C for assay of BAL cytokines later. The cell pellets were resuspended in FCS and smears were made on glass slides. The cells, after air drying, were stained with Wright-Giemsa (Biochemical Sciences Inc, Swedesboro, NJ) and their differential count was taken under a light microscope at 40 × magnification. Cell number refers to that obtained from lavage of both lungs/mouse.

### Lung parenchyma

Lung mincing and digestion was performed after lavage as described previously (Labarge S et al) with 100 u/ml collagenase for 1 hr at 37°C, and filtered through a 60# sieve (Sigma). All numbers mentioned in this paper refer to cells obtained from one lung/mouse.

### Lung histology

Lungs of other animals of same group were were fixed in 4% paraformaldehyde overnight at 4°C. The tissues were embedded in paraffin and cut into 5 μm sections. A minimum of 15 fields were examined by light microscopy. The intensity of cellular infiltration around pulmonary blood vessels was assessed by Hematoxylin and Eosin staining. Airway mucus was identified by staining with Alcian blue and Periodic Acid Schiff staining as described previously [14].

### Fluorescin-activated cell sorter (FACS) analysis

Cells from hemolysed peripheral blood (PB), bone marrow(BM), bronchoalveolar lavage (BAL), lung parenchyma (LP), spleen, mesenteric lymph nodes (MLN), cervical lymph nodes (CLN), axillary lymph nodes (LNX) and inguinal lymph nodes (LNI) were analyzed on a FACSCalibur (BD Immunocytometry Systems, San Jose, CA) by using the CELLQuest program. Staining was performed by using antibodies conjugated to fluorescin isothiocyanate (FITC), phycoerythrin (PE), allophucocyanin (APC), Peridinin Chlorophyll Protein (Per CP-Cy5.5) and Cy-chrome (PE-Cy5 and PE-Cy7). The following BD pharmingen (San Diego, CA) antibodies were used for cell surface staining: APC-conjugated CD45 (30F-11), FITC-conjugated CD3(145-2C11), PE-Cy5 conjugated CD4 (RM4-5), PE-conjugated CD45RC (DNL-1.9), APC-conjugated CD8(53-6.7), PE-Cy5 conjugated B220 (RA3-6B2), FITC-conjugated IgM, PE-conjugated CD19 (ID3), PE-conjugated CD21(7G6), FITC-conjugated CD23 (B3B4), APC-conjugated GR-1(RB6-8C5), and PE-conjugated Mac1(M1/70). PE-Cy5 conjugated F4/80 (Cl:A3-1(F4/80)) was obtained from Serotec Ltd., Oxford, UK. PE-conjugated anti-α4 integrin (PS2) and anti-VCAM-1(M/K-2) was from Southern Biotechnology, Birmingham, Ala. Irrelevant isotype-matched antibodies were used as controls.

### CFU-c assay

To quantitate committed progenitors of all lineages, CFU-C assays were performed using methylcellulose semisolid media (Stemgenix, Amherst, N.Y.) supplemented with an additional 50 ng of stem cell factor (Peprotech, Rocky Hill, N.J.) per ml. Next, 50,000 cells from bone marrow, 500,000 cells from spleen, 0.01 million cells from lung and BAL, and 10 μl peripheral blood were plated on duplicate 35-mm culture dishes and then incubated at 37°C in a 5% CO_2_-95% air mixture in a humidified chamber for 7 days. Colonies generated by that time were counted by using a dissecting microscope, and all colony types (i.e., burst forming units-erythroid [BFU-e], CFU-granulocyte-macrophage [CFU-GM], and CFU-mixed [CFU-GEMM]) were pooled and reported as total CFU-C. Total CFU-c per organ was calculated by extrapolating CFU-c against number of plated cells to the total number of cells in the organ.

### ELISA for cytokines

Th2 cytokines (IL-4 and 5) and TNFα and IFNγ in BAL and serum (previously frozen at -80°C) were outsourced to Linco plex Biomarker Immunoassays, Millipore. IL-13 was measured by Quantikine M kits from R&D Systems, Minneapolis, MN.

### OVA specific IgE and IgG1 in serum

Anti-mouse IgE (R35-72) and IgG1(A85-1) from BD Biosciences, San Diego, CA were used for measuring OVA specific IgE and IgG1 (in serum previously frozen at -70°C) respectively by standard ELISA procedures as previously described [14].

### Statistical analysis

Statistical differences among samples were tested by Student *t *test. *P *value less than 0.05 was considered statistically significant.

## Results

### Effect of gp91^phox ^deletion on development of composite asthma phenotype

**Figure 1:**
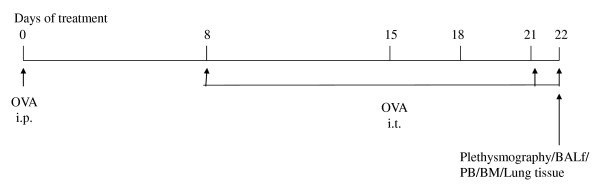
**Study design to generate acute allergic asthma phenotype in mice**: Mice were sensitized with OVA (100 μg) complexed with aluminium sulfate in a 0.2-ml volume, administered by i.p. injection on day 0 and later challenged with OVA On days 8 (250 μg of OVA) and on days 15, 18, and 21 (125 μg of OVA), (Pierce, Rockford, IL) by intra-tracheal instillation. Mice were immunized with OVA (100 μg) complexed with aluminium sulfate in a 0.2-ml volume, administered by i.p. injection on day 0. The control group received normal saline with aluminium sulfate by i.p. route on day 0 and 0.05 ml of 0.9% saline by i.t. route on days 8, 15, 18, and 21. They were sacrificed on d22. The abbreviations used are: i.p. intra-peritoneal; i.t. intra-tracheal; BAL, bronchoalveolar lavage; PB, peripheral blood.

Composite asthma phenotype developed completely and more aggressively in both gp91phox-/- and MMP12-gp91phox double knockout mice post OVA treatment as described in Figure 1. Inflammation in terms of total inflammatory migration (measured by FACS of surface marker expression and differential count of H&E stained cytospin smears) was increased (Table 1). In bone marrow, NOX-/- post OVA has 1.4-fold more cells (data not shown), in peripheral blood, 1.3-fold more cells, spleen had 1.3-fold more cells (data not shown), lung parenchyma had 1.8-fold more cells, and BALf: 2-fold more cells compared to post-OVA WT. Of note, 2.4-fold more PMNs, 1.96-fold more B lymphocytes, 5-fold more eosinophils in post-OVA NOX-/- and DKO compared to post-OVA WT BAlf was found. Overall systemic response, inflammatory recruitment from blood to inflammation in lung is more (Table 1).

**Table 1:** Number of cells (× 10^6^) of leucocyte subsets in peripheral blood, lung parenchyma and bronchoalveolar lavage fluid (BALf).

BLOOD	**Leukocyte subsets (× 10^6^) in blood, lungs and BAL fluid post OVA treatment**.								
	**Total** **WBCs**	**Lymphocytes**		**Monocytes**	**Neutrophils**	**Eosinophils**	**Basophils**		
		
		**CD3+**	**B220+**						

**WA**	11.9 ± 2.4	5.54 ± 1.17	3.08 ± 1.05	0.38 ± 0.185	3.648 ± 0.184	0.223 ± 0.014	0.066 ± 0.001		

**WO**	24.72 ± 6.41	7.31 ± 1.31	5.93 ± 1.17	1.62 ± 0.35	7.97 ± 1.14	0.74 ± 0.36	1.05 ± 0.02		

**NOXA**	14.2 ± 3.012	5.7 ± 1.865	2.99 ± 1.033	0.77 ± 0.053	4.56 ± 1.074	0.133 ± 0.043	0.028 ± 0.002		

**NOXO**	25.625 ± 4.063	6.186 ± 1.076	6.742 ± 0.964	2.424 ± 1.076	8.741 ± 0.064	1.061 * ± 0.004	0.556 ± 0.003		

**DKOA**	11.7 ± 2.76	4.69 ± 1.06	2.467 ± 0.96	0.63 ± 0.12	3.74 ± 1.27	0.106 ± 0.07	0.02 ± 0.001		

**DKOO**	27.56 ± 2.79	6.61 ± 1.49	7.27 ± 2.01	2.6 ± 0.47	9.39 ± 2.44	1.14 * ± 0.46	0.59 ± 0.14		

**Lungs**									

	**Total**	**Lymphocytes**		**Monocytes**	**Neutrophils**	**Eosinophils**	**Basophils/**	**M**ϕ	

	**WBCs**	**CD3+**	**B220+**				**Mast cells**		

**WA**	1.86 ± 0.543	0.179 ± 0.021	0.035 ± 0.002	0.826 ± 0.054	0.319 ± 0.054	0	0	0.497 ± 0.015	

**WO**	12.35 ± 3.96	4.87 ± 1.05	0.09 ± 0.01	0.37 ± 0.09	1.47 ± 0.43	2.22 ± 0.76	0.046 ± 0.012	3.09 ± 1.08	

**NOXA**	**5.49 #** ± 1.064	**0.446 #** ± 0.002	**0.113 #** ± 0.001	**2.593 #** ± 0.76	**0.892 #** ± 0.011	0	0	**1.489 #** ± 0.016	

**NOXO**	22.6 * ± 7.544	4.084 ± 1.075	1.29 * ± 0.643	3.171 * ± 1.075	4.337 * ± 1.074	4.102 * ± 1.066	0.089 * ± 0.002	5.682 ± 0.064	

**DKOA**	**3.78 #** ± 1.07	0.3 ± 0.14	0.07 ± 0.01	**1.78 #** ± 0.54	0.61 ± 0.02	0.001 ± 0.0001	0	**1.02 #** ± 0.29	

**DKOO**	21.66 * ± 408	3.89 ± 1.06	0.43 * ± 0.16	3.03 * ± 0.89	4.16 * ± 1.33	3.93 * ± 1.13	0.08 * ± 0.002	5.44 * ± 1.26	

**BAL fluid (2 lungs)**									

**WA**	0.8 ± 0.013	0	0	0.013 ± 0.004	0	0	0	0.787 ± 0.011	

**WO**	9.46 ± 0.89	1.64 ± 0.21	1.54 ± 0.021	1.26 ± 0.03	0.86 ± 0.01	4.26 ± 0.07	0.04 ± 0.002	0.56 ± 0.004	

**NOXA**	0.63 ± 0.018	0	0	0.014 ± 0.002	0	0	0	0.616 ± 0.003	

**NOXO**	16 * ± 3.159	2.04 ± 0.754	1.213 ± 0.003	2.08 ± 0.074	2.427 * ± 1.066	6.741 * ± 2.076	0.051 ± 0.001	1.568 * ± 0.72	

**DKOA**	0.865 ± 0.01	0	0	0.025 ± 0.002	0	0	0	0.84 ± 0.18	

**DKOO**	15.89 * ± 4.03	2 ± 0.86	1.22 ± 0.66	2.05 ± 0.75	2.415 *± 1.01	6.67 * ± 1.14	0.05 ± 0.003	1.55 * ± 0.63	

Number of cells (× 10^6^) of leucocyte subsets in peripheral blood, lung parenchyma and bronchoalveolar lavage fluid (BALf).									

		**T cells**	**B cells**	**Monocytes**	**Neutrophils**	**Eosinophils**	**Basophils**		

Lungs	WO	0.6	0.015	0.22	0.18	3	0.04		

	NOXO	0.6	0.19*	1.3	0.49*	3.8	0.16*		

	DKOO	0.58	0.05	1.1	0.44*	3.4	0.13*		

BALf	WO	0.22	0.25	0.77	0.107	5.75	0.03		

	NOXO	0.32	0.17	0.85	0.27*	6.3	0.09		

	DKOO	0.51	0.16	0.78	0.26*	5.8	0.08		

Total white blood cells were counted by a Beckman Coulter particle counter and subsets quantified using specific antibody staining in FACS and corroborated by morphological assessment by light microscopy and also by hemavet using the specifics of mouse. The table shows data averaged from three independent experiments ± SEM. * denotes p value < 0.05 compared to values from post-OVA wildtype mice and # denotes p value < 0.05 compared to alum treated wildtype. Abbreviations used are: WA = wildtype +alum, WO = wildtype + OVA, NOXA = gp91phox-/- + alum, NOXO = gp91phox-/- + OVA, DKOA = MMP12-gp91phox double knockout + alum, DKOO = MMP12-gp91phox double knockout + OVA. (n = 5/group). **Recruitment index: Inflammatory hematopoietic cells found in lungs and BALf expressed as a fraction of corresponding cells in circulation**. Number of cells (× 10^6^) in lungs and BALf were the numerator and the number of cells (× 10^6^) in circulating blood was the denominator in the fraction calculated to show recruitment index or inflammatory cells recruited from circulation to lung parenchyma and airways in response to the OVA allergic challenge. * denotes statistically significant change (p value < 0.01)

### Pulmonary function test

**Figure 2:**
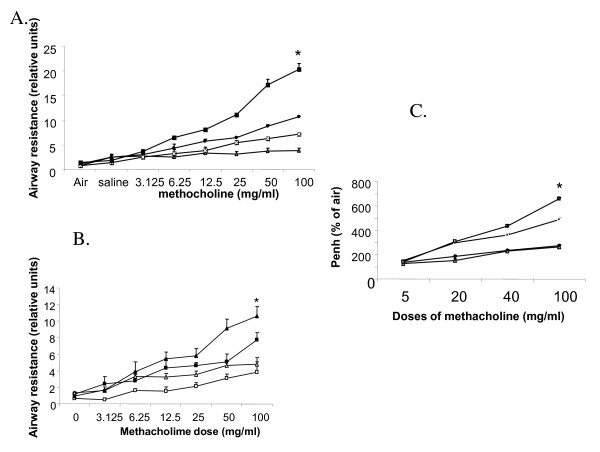
**Pulmonary function testing**. **A**. Airway resistance in WT vs. gp91phox-/-. Symbols used are white triangle WA (Wildtype alum), black circles WO (WT OVA), white cross in black box NOX (alum gp91phox-/-) and black box NOXO (OVA treated gp91phox-/-). Airway resistance obtained by invasive plethysmography show increased values in both gp91phox-/- and gp91phoxMM12 double knockout mice compared to wildtype post-OVA. Symbols used are open square WA, closed square WO, open triangle DKOA (alum double knockout), closed triangle DKOO (OVA treated DKO. On d 22, 24 h after the last intra-tracheal allergen (OVA) challenge invasive pulmonary mechanics were measured using a commercial plethysmography system (Model PLY4111 plethysmograph, MAX II amplifier and pressure transducer system, and Biosystem XA software, Buxco Electronics, Inc.) mice receiving aerosolized solutions of methacholine (0, 3.125, 6.25, 12.5, 25, 50, and 100 mg/ml in normal saline) via an AER 1021 nebulizer aerosol system (Buxco Electronics, Inc., Wilmington, NC) with 2.5-4 micron aerosol particle size generated by NEB0126 nebulizer head (Nektar Therapeutics, San Carlos, CA), and RL as calculated from measures of pressure and flow and expressed as cmH2O/ml/s was determined. Figures show Airway resistance (RL) values ± SEM of data obtained from 3 independent experiments (n = 5/group). **C**. Non-invasive plethysmography (expressed as Penh) was also assessed on d 22 in independent experiments with gp91phox-/- only and this co-related with the airway resistance values obtained by invasive plethysmography. Symbols used are black diamond WA, open triangle NOX, small black square WO, open large square NOXO: For non-invasive plethysmography, mice were challenged with increasing doses of aerosolized methacholine (0, 5, and 20, 40, and 100 mg/ml in normal saline) generated by an ultrasonic nebulizer (DeVilbiss Health Care, Inc., Somerset, PA) for 2 min. and the degree of bronchoconstriction was expressed as enhanced pause (Penh), a calculated dimensionless value that correlates with measurement of airway resistance, impedance, and intrapleural pressure. Penh is primarily independent of FRC, tidal volume, and respiratory rate since the ratio of measurements is obtained during the same breath and has a strong correlation with both airway resistance (Raw) measured directly in anesthetized, tracheotomized, and mechanically ventilated mice. *denotes p value < 0.01 compared to post-OVA wildtype values.

Pulmonary function test measured by both non-invasive plethysmographt and invasive plethysmography (Figure 2A-C) show 2.5-fold increase in Penh values compared to WT post-OVA at 100 mg/ml dose of methacholine. 1.89 and 1.35-folds increase was found in gp91phox-/- and DKO respectively at 100 mg/ml dose of methacholine compared to WT post-OVA by invasive plethysmography of anesthetized mice.

### Cellularity in bone marrow, blood, lungs and airways

**Figure 3:**
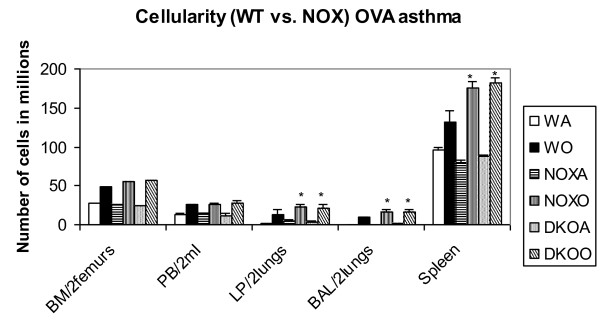
**Increase in total cell number in lung and BALf of knockout mice compared to post-OVA WT**. Cell number was counted using a Z1 particle counter from Beckman Coulter. Bone marrow cells (BM) was flushed out of two femurs, blood (PB) was obtained by infra-orbital bleeding and extrapolated to a 2 ml volume as the total volume of blood in a 20 gm mouse, from the volume of blood actually obtained, perfused lung was minced and digested with collagenase IV and single cell suspension made of both lungs, and brochoalveolar lavage fluid (BALf) was obtained from both lungs, and the cell numbers counted. The data shown have been derived from 3 independent experiments and expressed as mean values ± SEM. *denotes p value < 0.01 compared to post-OVA wildtype values. Abbreviations used are: WT = wildtype, NOX = gp91phox-/-, DKO = gp91phox-MMP12 double knockout, WA = WT+alum, WO = WT+OVA, NOXA = gp91phox-/-+alum, NOXO = gp91phox-/-+OVA, DKOA = gp91phox-MMP12 double knockout+alum, DKOO = gp91phox-MMP12 double knockout+OVA.

Cellularity was increased in post-OVA mice compared to saline treated mice. Figure 3 shows 1.8-fold and 1.7-fold increase in number of cells in lung parenchyma and BALF and 1.4-fold in spleen in both KO mice than WT. Recruitment index (Table 2) was increased in B cells, monocytes, PMNs, Eosinophils and basophils in both KO mice post-OVA compared to post-OVA WT.

**Table 2:** Clonogenic potential of progenitors

	PB/2 ml	BM/2femurs	LP/2lungs	BAL/2lungs	Spleen
WA	5371.6 ± 188.6	17034.5 ± 158.8	241.93 ± 11.8	3.15 ± 2.13	75858.9 ± 523.5
WO	16915.6 ± 219.1	57161.5 ± 161.9	11225.4 ± 155	5573.8 ± 41.6	188275.6 ± 361.1
NOXA	2343 ± 244.3	8293.3 ± 197.1	604.1 ± 125.8	59.9 ± 19.1	41774.2 ± 75.4
NOXO	9403.3 ± 382.2	40677.7 ± 220.4	6299.3 ± 195.9	5921.7 ± 41.63	219677.5 ± 426.8
DKOA	4591.6 ± 256.6	11783.1 ± 284.5	562.9 ± 183.7	32.9 ± 45.7	65974.3 ± 344.3
DKOO	9262.1 ± 342.7	41278.3 ± 361.7	7103.1 ± 201.6	6012.2 ± 59.7	197822.7 ± 388.7

**Decrease in clonogenic potential in bone marrow and lung of both KO mice post-OVA**. CFU (Colony Forming Units) as assay was done as described in Materials and Methods and assayed in duplicate petridishes per sample. The results shown are pooled from three independent experiments (n = 5/group) ± SEM. Abbreviations used are: BM, bone marrow; PB, peripheral blood, LP, lung parenchyma, BALf, bronchoalveolar lavage fluid. The total number of progenitors was extrapolated to the total number cells obtained from each tissue. Underlined numbers denote p value < 0.01 compared to post-OVA WT.

### Progenitors in bone marrow, blood, lungs and airways

**Figure 4:**
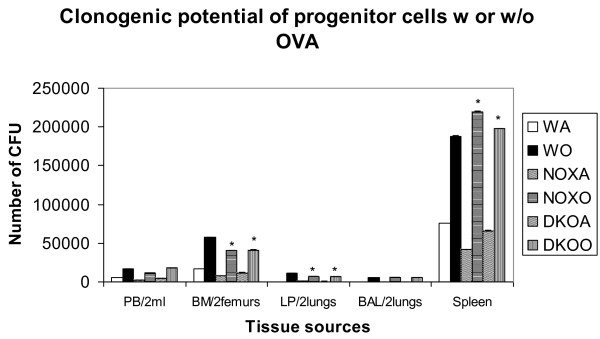
**Clonogenic potential was decreased in the bone marrow and lung progenitors of both knockout mice post OVA**. 10 ml heparinized whole peripheral blood (PB) obtained by infra-orbital bleeding of anesthetized mouse, 50,000 bone marrow cells (BMC)flushed out of the femurs, 1 million lung parenchyma (LP) cells digested by collagenase IV, 1 million cells from bronchoalveolar lavage fluid (BALf)and 500,000 cells from spleen was plated in 2 ml semi solid methyl cellulose medium and cultured for 7 d for PB, BM and spleen and for 14d for LP and BALf. Colony forming units (CFU) was counted manually on a Leica DMIL and an Olympus sZX12 inverted microscope. Results shown are pooled from three independent experiments (n = 5/group) ± SEM. * denotes p value < 0.01 compared to post-OVA wildtype values. Abbreviations used are: WT = wildtype, NOX = gp91phox-/-, DKO = gp91phox-MMP12 double knockout, WA = WT+alum, WO = WT+OVA, NOXA = gp91phox-/-+alum, NOXO = gp91phox-/-+OVA, DKOA = gp91phox-MMP12 double knockout+alum, DKOO = gp91phox-MMP12 double knockout+OVA.

Table 2 and Figure 4 shows decrease in number of colony forming units in post-OVA bone marrow and lung of both KO mice compared to similarly treated WT. Progenitor number was however somewhat upregulated in both KO groups post-OVA compared to Post-OVA WT.

### Inflammation in the lungs

**Figure 5:**
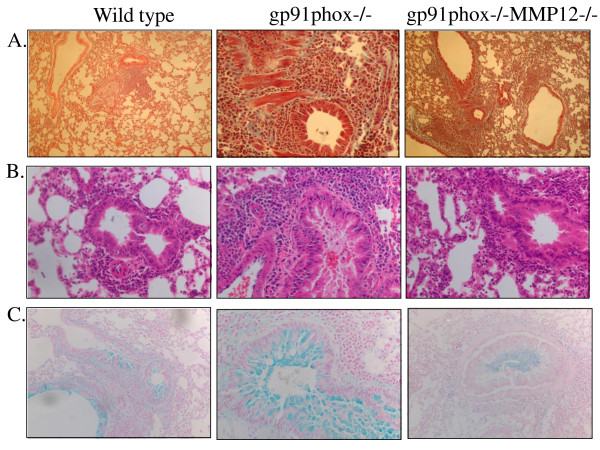
**Both knockout mice show increased inflammation and mucus secretion post OVA compared to WT**. Paraffin sections of lungs of OVA-treated mice were stained with A, Hematoxyline and eosin (10×), B. with Masson's Trichrome stain (20×), and C. Alcian blue counterstained with eosin (10×). Sections were viewed with a BX4l Olympus microscope and photomicrographs taken with a Nikon Spot digital camera. Inflammatory cell migration was more pronounced around airways in the OVA-treated knockout mice compared to control.

Histopathology showed a marked increase in peribronchiolar and perivascular infiltration of inflammatory cells post-OVA in both KO mice compared to control (Figure 5A-C). The photomicrographs show a marked increase in both number of inflammatory cells and mucus secretion in airways.

### Airway goblet cell metaplasia

**Figure 6:**
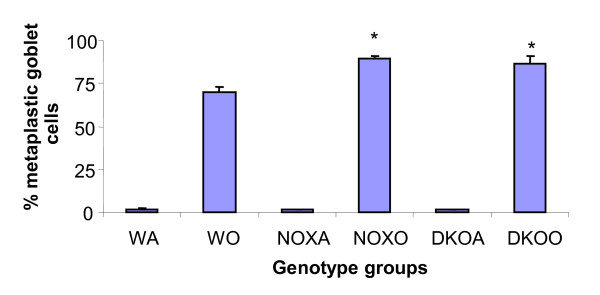
**Increase in percent alcian blue positive cells (goblet cell metaplasia) in gp91phox-/- and gp91phox-MMP12 double knockout (DKO) post-OVA compared to wildtype (WT) post-OVA**. Metaplastic goblet cells were counted as the blue cells stained positive by alcian blue. Percent metaplastic goblet cells is calculated from the total number of cells counted around each airway. Abbreviations used are: WT = wildtype, NOX = gp91phox-/-, DKO = gp91phox-MMP12 double knockout, WA = WT+alum, WO = WT+OVA, NOXA = gp91phox-/-+alum, NOXO = gp91phox-/-+OVA, DKOA = gp91phox-MMP12 double knockout+alum, DKOO = gp91phox-MMP12 double knockout+OVA. The results shown are pooled from 3 independent experiments. Counts were taken and averaged over 10 high power fields. Slides were counted on a Spencer AO light microscope at 40 × magnification. Results are expressed as percent ± SEM. n = 5 animals/group. * denotes p value < 0.01 compared to post-OVA wildtype values.

1.27-fold and 1.22-fold increase in percent metaplastic goblet cells (Figure 6) was measured by counting under light microscope, the blue mucus laden cells around airways vs. the pink squamous cells which do not show metaplasia.

### Th2 cytokine release in airway

**Figure 7:**
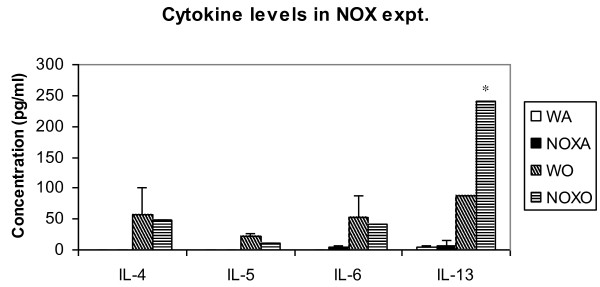
**Cytokine concentration in BALf**. The concentration of cytokines in BALf was measured by outsourcing to Linco by multiplexing technique in a luminometer. Data expressed here are mean ± SEM. n = 5/group. While all other Th2 cytokine levels were comparable to WT+OVA, IL-13 concentration was increased 2.7-fold over post-OVA WT values. Abbreviations used are: WT = wildtype, NOX = gp91phox-/-, DKO = gp91phox-MMP12 double knockout, WA = WT+alum, WO = WT+OVA, NOXA = gp91phox-/-+alum, NOXO = gp91phox-/-+OVA.

Cytokine concentrations present in the BALf was measured by ELISA. Figure 7 shows 2.75-fold increase in IL-13 level in gp91phox-/- mice post-OVA compared to WT post-OVA. All other Th2 cytokines showed values similar to post-OVA WT BALf. **Data **shows that actual mRNA upregulation was 1.4-fold for IL-4 gene and 1.9-fold for IL-13 genes which are Th2 specific. There was also upregulation in IL-1α, IL-10 and IL-12α, the dignificance of which is not clear at this point. cells secreting these are T cells, macs and some epithelial cells. Overall, IL-4: NOX-/- post OVA has 1.2-fold more protein and 2-fold more mRNA; IL-5: NOX-/- post OVA has 2-fold more protein and 2.8-fold more mRNA; IL-13: NOX-/- post OVA has 3-fold more protein and 5.6-fold more mRNA. Therefore, both by protein concentration and mRNA expression, Th2 cytokines show manifold increase in gp91phox-/- post-OVA compared to WT.

### Functionality of B cells

**Figure 8:**
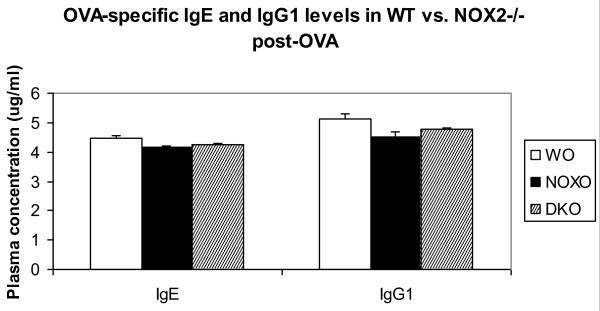
**Plasma concentrations of OVA-specific IgE and IgG1 are comparable between Wt and KO mice post-OVA**. OVA-specific IgE and IgG1 were measured by standard ELISA using a capture and a detection antibody for each of the immunoglobulins. Data showed represents mean of values pooled from three independent experiments (n = 5/group). Abbreviations used are: WT = wildtype, NOX = gp91phox-/-, DKO = gp91phox-MMP12 double knockout, WO = WT+OVA, NOXO = gp91phox-/-+OVA, DKOO = gp91phox-MMP12 double knockout+OVA. Levels of OVA-specific cytokines in the alum treated corresponding control groups was undetectable.

B cell function was tested by measuring OVA-specific IgE and IgG1 in post-OVA WT and the two KO mice. Figure 8 shows that the values are comparable.

## Discussion

Phagocytes play a critical role in host defense by producing reactive oxygen species against invading microorganisms. One of the most important enzymes in producing microbicidal oxidants is the superoxide (O2 2)-generating NADPH oxidase.1 The NADPH oxidase is a multicomponent enzyme complex whose redox center is a membrane-associated flavocytochrome *b*558 heterodimer composed of gp91*phox *and p22*phox*. In addition, three cytosolic oxidase subunits, p47*phox*, p67*phox*, and a low molecular weight GTP binding protein Rac, are required for high level production of O2 2. In resting phagocytes, the dormant oxidase is unassembled. However, upon phagocyte activation, the active oxidase complex is rapidly formed by translocation of the cytosolic oxidase components to the plasma membrane via interactions with the cytochrome.2 Subsequently, electrons are transferred from cytosolic NADPH to molecular oxygen (O2) at the external face of the membrane to generate superoxide anion [15].

In activated neutrophils, a membrane-bound NADPH-dependent oxidase generates high levels of superoxide, a process traditionally called "respiratory burst". In unstimulated neutrophils, components of the NADPH oxidase complex are separated in cytosol (p40*phox*, p47*phox*, p67*phox*, Rac2) and membrane compartments (flavocytochrome *b558*, Rap1A). During phagocytosis, the cytosolic components translocate to the plasma and/or phagosome membrane and associate with flavocytochrome *b558*, a transmembrane heterodimer comprised of gp91*phox *(Nox2) and p22*phox*, thereby forming the active oxidase. The oxidase transfers electrons from cytosolic NADPH to intraphagosomal molecular oxygen, thus producing superoxide. Superoxide anion is short-lived and dismutates rapidly to hydrogen peroxide and forms other secondary products, such as hypochlorous acid, hydroxyl radical, and singlet oxygen, which are effective microbicidal compounds [16].

Briefly, deletion of gp91^phox ^results in enhancement of composite asthma phenotype in mouse. Double deletion of gp91phoix and MMp12, a critical enzyme for phagocyte associated inflammation results in no alteration of the phenotype generated in the single deletion of gp91phox-/-. Overall inflammation is robust. Both naïve and allergen challenged KO mice show statistically significant exacerbation of Th2 inflammation. All the classical features of a robust acute allergenic asthma phenotype are significantly amplified in the KO mice showing that NADPH oxidase, which is essentially an extra-cellualr matrix associated enzyme expressed by phagocytes, which by definition may denote a non-specific inflammatory response or at the best an inflammatory phenomenon associated with lung remodeling in chronic asthma, surprisingly may also have a regulatory role in the sensitization or priming stage. This is no more apparent than in the level of circulating and tissue bound progenitors which show a clear mobilization denoted by the dearth of stem as well as mature cells in bone marrow. Because of the absence of gp91phox, the cells seem to have less of an adherence to the stroma and migrate out of their niche. The cellular traffic out of bone marrow (CFU in femur was assessed) also shows a more robust lympho- and eosinophilopoiesis. Does gp91phox have any known role in hematopoiesis or granulocytopoiesis? [16] says that NADPH essentially increases neutrophil and eosinophil synthesis and migration to inflammatory tissues. If gp91phox is imagined to have a regulatory role in development of the asthma phenmotype, MMP12 seems to have a similar if not synergistic effect. These molecules are however not necessary for migration, but are critical for PMA induced proliferation and MCP-1 induced chemotaxis. The overall Th2 response was enhanced possibly due to a lack of control over T cell: APC cross-talk in the KO mice as shown by MLR.

The results described above indicate that gp91phox-/- mice respond to OVA in a more exaggerated fashion compared to WT post-OVA, in terms of total number of cells migrated to lung (1.8 folds) and BALf (1.7 folds), although total number of cells in bone marrow (obtained from two femurs) and that in circulating peripheral blood were comparable (Table 1). Expressed as a fraction of circulating cells recruited into lungs and BALf in response to allergic immune response, both knockout mice seem to show similar trends. Cell subsets for which recruitment index is more than 1, indicate a cumulative effect where both cells from circulation as well as resident cells normally present in the pulmonary milieu in surveillance, seem to be equally important. Recruitment of B cells, monocytes, neutrophils and basophils are increased in lungs of both knockout mice compared to post-OVA wildtype while that of T cells, neutrophils and basophils in BALf are increased in the knockout vs. the OVA-treated wildtype (Table 1). In gp91phox-/- progenitors in BM decrease by 28.8% while in double knockout, it decreases by 27.9% compared to the values in WT post-OVA. In lung, the decrease in progenitor number was 1.8-fold and 1.5-fold respectively. NOX2 and DKO mice both show spontaneous pulmonary inflammation. In naïve mouse, this may account for the decrease in cell number in femur and lungs (due to rapid and uncontrolled mobilization out of the niche and out of the parenchyma into the interstitium respectively. Spleen, because of this constant and uncontrolled inflammatory background naturally becomes inflamed and shows an increase in size and cell number. Under treatment with allergen, this scenario gets further aggravated. Femur (bone marrow) has been known to be the main pool of progenitors for granulopoiesis (PMNs and eosinophils). Cells from the lung parenchyma, on the other hand, need to migrate into the interstitium for Th2 category inflammation in the lung tissue. These may account for dearth of progenitors and mature cells in these tissues while spleen registered a higher than normal cell number anyway in these mice and under a heightened condition of inflammatory exacerbations, shows a higher reserve despite this limitation.

My work [17-22] in conjunction with other work in the public domain using genetic knockout models of mice in a preclinical set up to study molecular roles of deleted molecules in the pathogenesis of acute allergic asthma has revealed the following. The alternately delicate and robust interplay of sensitization-priming vs. fully activated state of cells play a crucial role in the final translation of the pathogenesis status of the animal. In humans Chronic granulomatous disease (CGD) where is a group of inherited disorders in which phagocytes are unable to generate superoxide due to genetic defects in any 1 of 4 essential NADPH oxidase components. Mutations in the X-linked gene for gp91phox, the large subunit of the flavocytochrome b558 heterodimer, account for the majority of CGD. Patients with CGD develop recurrent, often life-threatening bacterial and fungal infections due to impaired microbicidal oxidant generation by the patient's phagocytes. ROS are also important in inducing in schemia-induced lung angiogenesis. [23] gp91^phox^, expressed in CHO (Chinese hamster ovary) cells, functions as a voltage-dependent proton channel. [24] gp91phox was also found to regulate adaptive immune response at the level of both T cells and APCs [25].

Activation of NADPH oxidase represents an essential mechanism of defense against pathogens. Dendritic cells (DC) are phagocytic cells specialized in Ag presentation rather than in bacteria killing. Human monocyte-derived DC were found to express the NADPH oxidase components and to release superoxide anions in response to phorbol esters and phagocytic agonists. The NADPH oxidase components p47*phox *and gp91*phox *were down-regulated during monocyte differentiation to DC, and maturation of DC with pathogen-derived molecules, known to activate TLRs, increased p47*phox *and gp91*phox *expression and enhanced superoxide anions release. Similar results were obtained with plasmacytoid DC following maturation with influenza virus. In contrast, activation of DC by immune stimuli (CD40 ligand) did not regulate NADPH oxidase components or respiratory burst. NADPH oxidase-derived oxygen radicals did not play any role in DC differentiation, maturation, cytokine production, and induction of T cell proliferation, as based on the normal function of DC generated from chronic granulomatous disease patients and the use of an oxygen radical scavenger. However, NADPH oxidase activation was required for DC killing of intracellular *Escherichia coli*. It is likely that the selective regulation of oxygen radicals production by pathogen-activated DC may function to limit pathogen dissemination during DC trafficking to secondary lymphoid tissues [26]. Reactive oxygen species (ROS) play an important role in regulating vascular tone and intracellular signaling [27].

In summary, the work presenetd in this communication describes in detail, the phenotype expressed in two genotype knockout mice, gp91phox-/- and gp91phox, MMP-12 double knockout. The data obtained indicates clearly the following:

1. All aspects of acute allergic asthma are manifested in an exaggerated manner in the knockout mice vs. the WT, viz. cell recruitment is more, inflammatory cell recruitment,, specifically myeloid cells in increased; T cells are also recruited not only in greated numbers but are also directly involved in the disease pathogenesis by clearly increasing TH2 cytokines, antigen-specific IgG and hypertrophy and hyperplasia in goblet cells of the lung. So these molecules seem to have regulatory roles in (a) cell priming, (b) direct cell activation, and (c) cell recruitment (which may include synthesis, mobilization and homing out of their niches) and by extrapolation, dendritic cells, phagocytes (macrophage and neutrophils) and T cells in the afferent arm of the disease pathology and eosinophils, B cells as denoted by Ig sequestration and probably endothelial and smooth muscle cells of the lung as well, in the efferent arm (responsible for exacerbations) may be defined as components under direct regulation of these two molecules (gp91phox and MMP-12).

2. While gp91phox and MMP12 deletion affects the mature cell polulation thus, the progenitor numbers are also similarly affected. This possibly alludes to greated synthesis in bone marrow or adult tissue reserves as well as enhanced mobilization. This is an important finding as it provides a critical link between the actual increase in cell number and a decreased adhesion to stroma thereby setting the stage of more efficient mobilization. While gp91phox and MMP12 interaction have been known to orchestrate ROS activity and tissue damage during inflammtion, there was no information regarding the upstream implication between this manifestation of innate immunity and the actual mechanistic phenomenon encouraging cells to actually migrate in larger numbers and more quickly to tissue spaces. Whether this also aids specific tissue homing is unknown at the present time and needs more work in this model.

3. The function of gp91phox may be primary in these regulatory cascade and MMP-12 may come in later. However, based on the data presented in this study, they seem to have synergistic or at best redundant, but definitely not additive roles.

## Abbreviations

OVA: ovalbumin; BM: bone marrow; PB: peripheral blood; BALf: bronchoalveolar lavage fluid; LP: lung parenchyma; AHR: airway hyper-reactivity/responsiveness; i.t.: intra-tracheal; i.v.: intravenous; i.p.: intraperitoneal; H&E: Hematoxylin and Eosin; Penh: enhanced pause; WBP: whole body plethysmography; KO: knockout; ROS: reactive oxygen species; NOX: NADPH oxidase.

## Competing interests

The authors declare that they have no competing interests.

## Authors' contributions

ERB conceptualized, designed and performed the experiments, evaluated the data, and wrote this manuscript. WRH provided the fund and lab space and contributed with inputs during analyses and interpretation of the results. All authors read and approved the final manuscript.
